# Biofilms on Indwelling Artificial Urinary Sphincter Devices Harbor Complex Microbe–Metabolite Interaction Networks and Reconstitute Differentially In Vitro by Material Type

**DOI:** 10.3390/biomedicines11010215

**Published:** 2023-01-14

**Authors:** Glenn T. Werneburg, Daniel Hettel, Ava Adler, Sromona D. Mukherjee, Scott D. Lundy, Kenneth W. Angermeier, Hadley M. Wood, Bradley C. Gill, Sandip P. Vasavada, Howard B. Goldman, Raymond R. Rackley, Daniel A. Shoskes, Aaron W. Miller

**Affiliations:** Department of Urology, Glickman Urological and Kidney Institute, Cleveland Clinic Foundation, Cleveland, OH 44195, USA

**Keywords:** artificial urinary sphincter, incontinence, intrinsic sphincteric deficiency, neurogenic lower urinary tract dysfunction, neurogenic bladder, AMS800, biofilm, infection, post-prostatectomy incontinence, silicone

## Abstract

The artificial urinary sphincter (AUS) is an effective treatment option for incontinence due to intrinsic sphincteric deficiency in the context of neurogenic lower urinary tract dysfunction, or stress urinary incontinence following radical prostatectomy. A subset of AUS devices develops infection and requires explant. We sought to characterize biofilm composition of the AUS device to inform prevention and treatment strategies. Indwelling AUS devices were swabbed for biofilm at surgical removal or revision. Samples and controls were subjected to next-generation sequencing and metabolomics. Biofilm formation of microbial strains isolated from AUS devices was reconstituted in a bioreactor mimicking subcutaneous tissue with a medical device present. Mean patient age was 73 (SD 10.2). All eighteen artificial urinary sphincter devices harbored microbial biofilms. Central genera in the overall microbe–metabolite interaction network were *Staphylococcus* (2620 metabolites), *Escherichia/Shigella* (2101), and *Methylobacterium-Methylorubrum* (674). An *rpoB* mutation associated with rifampin resistance was detected in 8 of 15 (53%) biofilms. *Staphylococcus warneri* formed greater biofilm on polyurethane than on any other material type (*p* < 0.01). The results of this investigation, wherein we comprehensively characterized the composition of AUS device biofilms, provide the framework for future identification and rational development of inhibitors and preventive strategies against device-associated infection.

## 1. Introduction

The artificial urinary sphincter (AUS) is an effective treatment option for urinary incontinence. It may be used for incontinence due to intrinsic sphincteric deficiency associated with neurogenic lower urinary tract dysfunction (NLUTD), or for stress incontinence following prostatectomy. The AUS device itself generally consists of a urethral cuff, the pump, and the reservoir. AUS devices are subject to infection, and about 3% will require surgical removal [1]. In the population with NLUTD in whom AUS devices are implanted for intrinsic sphincteric deficiency, infection rates may be higher due to the higher incidence of clean intermittent catheterization and associated erosion risk [2,3]. Antibiotic coating and impregnation has been used in the AUS for over a decade to reduce infection rates [1]. One commercially available coating includes a combination of rifampin and minocycline. Whether any benefit of this coating outweighs its cost remains to be determined [1,4]. Even in the absence of infection, there is evidence that AUS devices harbor microbes. For example, in a series of 80 patients in whom AUS devices were explanted for noninfectious reasons, 31 (39%) had a positive culture from a device component [5].

Incontinence devices in various stages of clinical use and development are composed of different material types. The AMS 800 artificial urinary sphincter remains the most commonly used AUS device, and is composed of silicone elastomer [6]. The Tape Mechanical Occlusive Device (TMOD, GT Urological LLC, Minneapolis, MN, USA) is a one-piece device made of microporous expanding polytetrafluoroethylene (PTFE) [7]. The Pro-ACT device (Uromedica, Inc., Plymouth, MN, USA) consists of two silicone balloons and a titanium post [8]. The periurethral constrictor continence device (Silimed, Rio de Janeiro, Brazil) includes a polyurethane foam coat [9,10]. Given the incidence of infection associated with medical devices, including the AUS, especially when implanted in the context of NLUTD, there is great interest in the identification and design of optimal materials and coatings to reduce infection risk.

To inform future approaches to prevent or treat device-associated infection, we sought to characterize the biofilm composition of the AUS device and compare biofilm formation of isolated bacterial species from indwelling AUS devices on material types used in implanted incontinence devices. We set out to identify biofilm microbial genera, metabolites, microbe–metabolite interaction networks, and antibiotic resistance genes. We tested relevant medical device materials in vitro using an assay designed to mimic human tissue with an indwelling medical device. We hypothesized that AUS devices would consistently harbor detectable bacterial and metabolite profiles, and that there would be differential biofilm formation both by bacterial strain and device material type. 

## 2. Materials and Methods

### 2.1. Subjects and Demographics

Patients scheduled for artificial urinary sphincter removal or revision for any indication were identified, and informed consent was obtained via IRB-approved protocol (20-415). Subjects who were 18 years of age or older were included in the study. Individuals who were not able to provide informed consent were excluded. Eighteen subjects were included. Average age was 73 (standard deviation 10.2) and cohort demographics are indicated in Table 1. All subjects from whom samples were obtained were male. Body mass index was 28.9 kg/m^2^ and 2 of 18 patients had diabetes mellitus. Indications for device removal included device malfunction (13), cuff erosion (3), device-associated infection (1), and lack of clinical efficacy (1). The average device indwelling time was 6.4 (SD 4.8) years. The average operative time at device placement was 115 (SD 47) minutes. 

### 2.2. Sample Processing

Before surgically accessing the device, the subcutaneous tissue was swabbed for use as a negative control. Then, the first-accessed portion of the AUS device was swabbed with three standard culture swabs to sample the device biofilm (swab 1: 16S next-generation sequencing; swab 2: metabolomics; swab 3: standard culture). Care was taken to avoid contamination from the remainder of the operative field during swab sample collection. Sample collection was performed under direct supervision by a study investigator. Demographics and patient factors were captured prospectively. 

Sample swabs were maintained at 4 °C for no more than 4 h following collection, and then transferred to −80 °C. The samples, together with negative and positive controls, were then analyzed using 16S next-generation sequencing, untargeted metabolomics, and culture-based assays to determine microbial and metabolic composition and diversity, and their association with patient clinical factors. 

Standard, validated approaches were used to extract DNA and metabolites as previously described [11,12,13]. For DNA extraction, bacteria were dislodged from the first swab in a sterile PBS solution with agitation. The cell lysis protocol employed bead-beating, piston-driven lysis, and proteinase K treatment. Then, DNA was attached to magnetic beads in a salt solution, and subsequently eluted in buffer. 

The second swab was similarly agitated in sterile PBS for preparation for untargeted metabolomics. Each metabolomics sample was diluted in acetonitrile solution containing the internal standards 4-nitrobenzoic acid (Acros Organics, Fair Lawn, NJ, USA), and debrisoquine (Santa Cruz Biotechnology, Dallas, TX, USA) [11,14]. To precipitate proteins, centrifugation was performed and the supernatant was recovered and stored at −80 °C until the time of analysis [15].

The third swab was streaked on Blood, CNA, Chocolate, and MacConkey agar plates for culture-based assays. The media were chosen to reflect the conditions used by the authors’ institution’s clinical microbiology laboratory, but with increased inoculum (swabs directly plated without dilution) and incubation time. Plates were incubated at 37 °C for 5 days. Next, colonies were transferred to new plates for isolation, and isolated colonies were transferred to liquid BHI culture. Bacteria were grown in culture to logarithmic or stationary phase. DNA was then extracted from the culture as described for the first swab. Glycerol stocks of culture were prepared and stored at −80 °C until the time of analysis.

### 2.3. Sequencing

DNA samples from the first swab were next submitted to the authors’ institution’s Microbial Sequencing and Analytics Core, where high-throughput 16S rRNA gene sequencing was performed using an Illumina iSeq. DNA amplification was performed using PCR with 515F and 806R primers designed to target the V4 hypervariable region of the 16S gene [13]. DNA concentration was determined and normalized, and library prep was performed with the Illumina Nextera XT library kit. Runs were performed to generate 150 bp, paired-end sequences. Positive controls of duplicate DNA standards (Zymobiomics, Irvine, CA, USA) were included. Negative controls were DNA reagents that were subjected to the entire protocol workflow, sterile water, and a PCR negative. 

Extracted DNA from culture bacterial isolated from the third swab was PCR-amplified using 27F and 1492R primers. Sanger sequencing by the genomics core of our institution was performed, and sequences were taxonomically assigned using BLAST.

### 2.4. Antibiotic Resistance and Biofilm Gene Detection

The antibiotic resistance genes *ampC* (penicillin-resistance-associated), *sul2* (sulfonamide-resistance-associated), *tetA* (tetracycline-resistance-associated), and an *rpoB* gene mutation (rifampin-resistance-associated) were detected using reverse transcriptase PCR (rtPCR) using a universal bacterial primer as a standard, as previously described [16]. Similarly, the *fimH* gene (biofilm-formation-associated) was detected. Previously reported detection primers were used as described [17,18,19,20,21]. 

### 2.5. Metabolomics

Fractions containing metabolites, which were prepared as detailed above, were processed in the Metabolomics Core at the authors’ institution, and metabolomics assays were performed as described [11,15]. Briefly, liquid chromatography/tandem mass spectrometry (LC-MS-MS) was used, and external standards were added to the samples. Negative controls were extraction buffers without sample, with standards included. Controls were run at four points along the sample queue to ascertain the technical consistency in metabolite quantification. Sample injections were performed onto the Vanquish UHPLC system coupled to the Q Exactive HF hybrid quadrupole–orbitrap mass spectrometer (Thermo Scientific, Waltham, MA, USA). The positive and negative electrospray ionization modes in the mass range 50–750 Da were used. Deconvolution of raw data was performed with XCMS software [22]. Ions detected were normalized to creatinine. Further analysis was performed using Metabolyzer software [23]. Quantification of concentration was performed through comparison to the added internal standards. Ionization, molecular mass, and retention time (*m*/*z*) were used to identify metabolites. Comparison to metabolites present in KEGG, HMDB, LIPIDMAPS, and BioCyc databases was used for putative identity assignment [24,25,26,27].

### 2.6. Bioinformatics

16S sequencing data were obtained as above and processed in R statistical packages unless otherwise noted. Bioinformatic analyses of 16s data were performed as described in [13]. Quality control, bimera removal, and amplicon sequence variants (ASV) assignment were performed using Dada2 [28]. ASV assignment was performed using a combined database of Silva 138 SSURef [29] and NCBI 16S Rrna [30]. ASV alignment was performed via MSA [31], and ASVs were arranged into a maximum-likelihood phylogeny in phangorn [32]. The resulting phylogenetic trees were combined with ASV tables, and this was merged with sample data and loaded into PhyloSeq, and taxa assigned to chloroplasts, mitochondria, or eukaryotes were removed [33]. 

The sequencing depth threshold to adequately capture microbial diversity was calculated in Vegan with rarefaction analysis [34]. Samples below the depth threshold were removed from further analysis. Decontam was used for removal of contamination with negative controls as the source [35]. The count table containing the remaining high-quality reads following decontamination was normalized using DESeq2 [36], which executes a negative binomial Wald test, minimizing differences based on sequencing depth while maintaining rare taxa. Alpha-diversity was next calculated from the normalized table using the phylogenetic diversity metric in Phyloseq. This metric quantifies the number of unique phylogenetic groups per sample. Beta-diversity was next calculated as a weighted UniFrac distance [37]. The weighted UniFrac metric quantifies microbial community differences based on the presence/absence of phylogenetic groups, along with their relative abundance. PERMANOVA was used to conduct beta-diversity statistical analyses after 999 permutations. For alpha-diversity, outliers from a run that contained only two samples, and thus with very high sequencing depth, were removed. The remainder of the data were analyzed using the paired *t*-test with Holm’s correction, where applicable. Sequences from isolated bacteria were mapped against the high-throughput sequencing data to validate that the high-throughput sequencing data originated from viable bacteria in the biofilms.

Using the BER package, normalized metabolite concentrations from positive and negative electron spray ionization modes were batch-corrected [38]. This package uses a linear regression model to locate and scale batch effects, while maintaining treatment effects when present. The algorithm has been previously validated for untargeted metabolomic data derived from urinary samples [15] and plasma samples [39]. PERMANOVA was used to conduct statistical analyses of dissimilarity matrices with 999 permutations in the VEGAN package [34].

Integration of 16S rRNA and untargeted metabolomic data was performed by the calculation of all pairwise microbe–metabolite Pearson correlations. Correlations > 0.4 with false-discovery-corrected *p*-values < 0.05 were used in subsequent analyses. Network visualization was performed using Cytoscape [40].

### 2.7. Continuous-Flow Stir Tank Bioreactor Biofilm Assays and Scanning Electron Microscopy

A continuous stir-tank CDC bioreactor was used to reconstitute and quantify biofilm formation in vitro. Bacteria isolated from AUS devices were incubated in the bioreactor in the presence of a series of medical device materials as previously described [41]. Based upon clinical relevance, viability on culture, and centrality in microbe–metabolite interaction networks, five microbial species isolated from AUS devices were analyzed for biofilm formation in vitro. Silicone, PTFE, polyurethane, polycarbonate, and titanium materials were included. The bioreactor is a large culture tank with controlled volume, temperature, turbulence, and flow rate, designed to mimic those of human tissue with an indwelling medical device present. The culture tank was filled with BHI media and inoculated with a microbial strain preculture grown to logarithmic or stationary phase. The negative control was performed identically, but with sterile BHI buffer without microbial strain. All negative controls exhibited no growth. Bioreactor temperature was 37 °C and flow rate was 1 mL/min. Each strain was incubated in the bioreactor with the device material coupons for 72 h. Device material types for a given microbial strain were included in triplicate. Following the 72 h incubation period, coupons were retrieved, rinsed in PBS, and dried by capillary action. Next, biofilm adherent to coupons were resuspended in PBS solution using a combination of scraping of all surfaces and agitation. This protocol was applied consistently across coupons to ensure consistent extraction and resuspension. Resuspension solutions were then plated in a series of tenfold dilutions on BHI agar plates and incubated overnight. Plate colony counts were then conducted using standard approaches, correcting for the dilutions. Differences among materials and strains were analyzed using ANOVA and Bonferroni t-tests. *p*-values < 0.05 were considered statistically significant.

A subset of coupons was analyzed using scanning electron microscopic analysis. After bioreactor incubation as above, coupons were first rinsed in PBS solution and then fixed in 4% formalin solution. Coupons were then serially dehydrated in ethanol and osmium tetroxide. Coupons were then submitted to the Imaging Core at the authors’ institution for processing. Fixed, dehydrated samples were mounted and gold-sputter-coated. Image acquisition was performed using a Zeiss Sigma VP scanning electron microscope [41].

## 3. Results

### 3.1. Biofilm Microbial Composition

**Figure 1:**
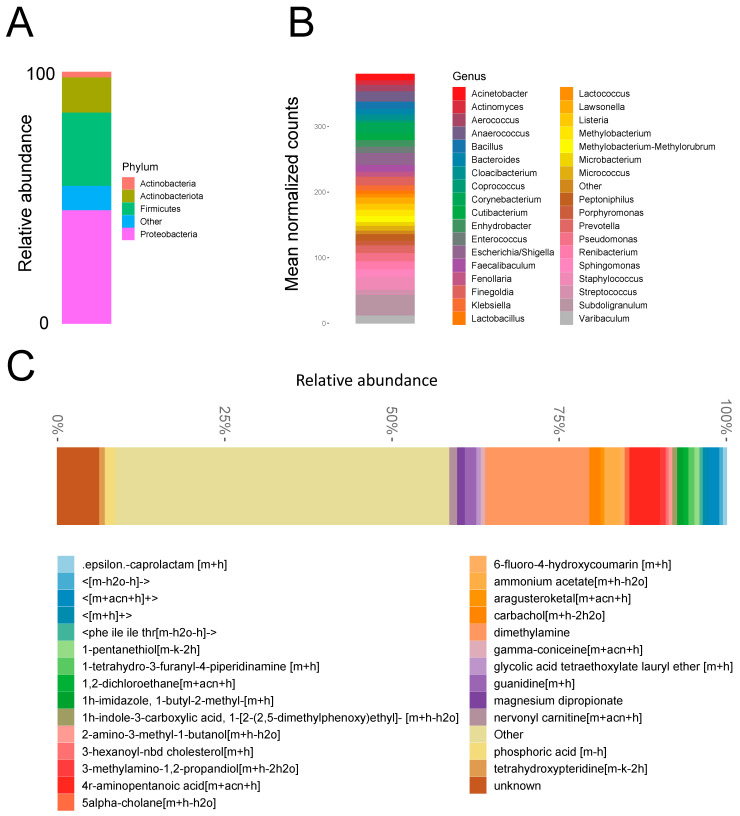
**Artificial urinary sphincter device biofilm microbe and metabolite composition.** Relative abundance of microbial phyla (**A**) and normalized counts of microbial taxa (**B**) on device biofilms as detected by 16S sequencing. (**C**) Relative abundance of metabolites as detected using mass spectroscopy (LC-MS-MS). See also Supplementary Data Set.

**Figure 2:**
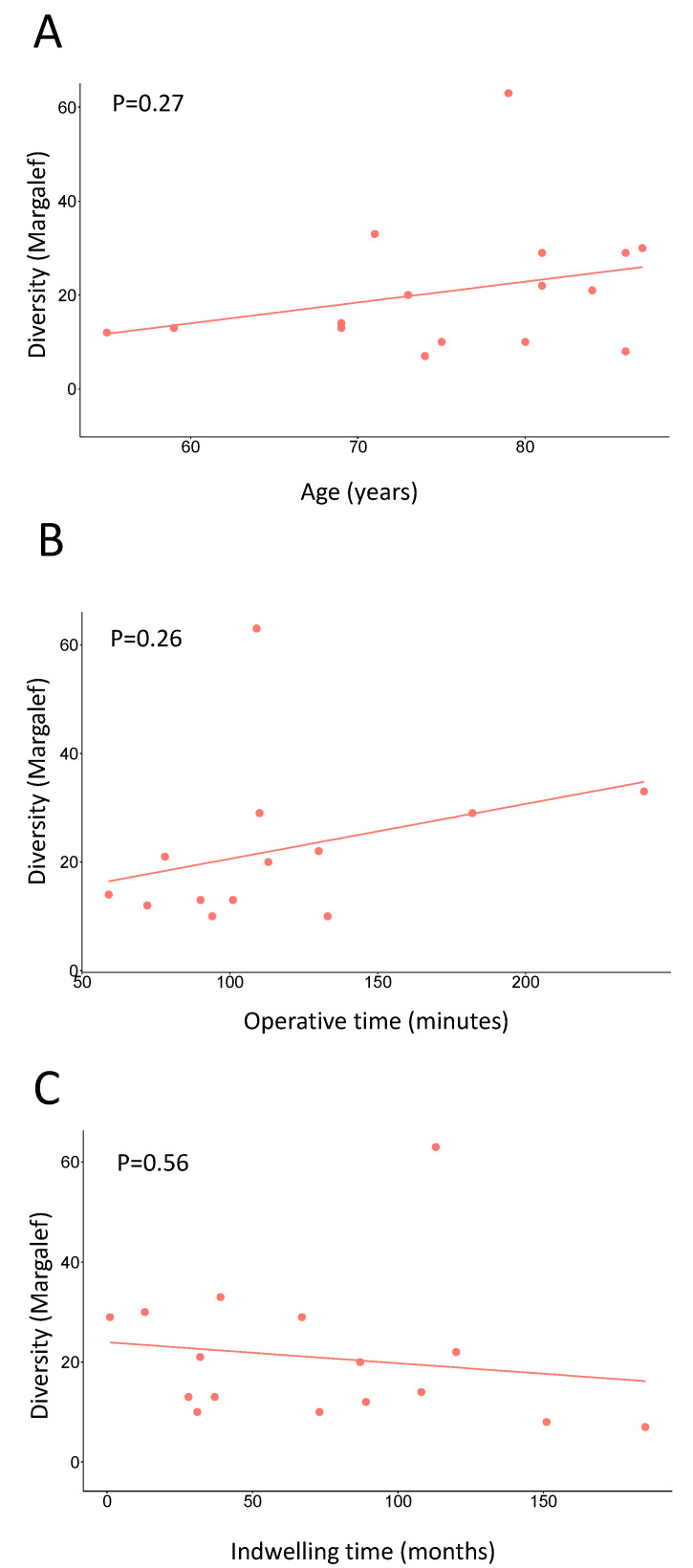
**Microbial diversity of artificial urinary sphincter device biofilms by patient factors.** Microbial diversity was similar by patient age (**A**), operative time at index procedure (**B**), and device indwelling time (**C**). Alpha diversity is indicated. *p*-values are indicated in their respective panels.

Eighteen AUS devices were included in the study. Microbial biofilms were detected on all 18 analyzed devices. Rarefaction analysis demonstrated that 18 of 18 prosthesis samples adequately captured microbiota diversity. The predominant microbial phyla detected by next-generation sequencing were Proteobacteria, followed by Firmicutes (Figure 1A). Detected genera are shown in Figure 1B and include the common clinical pathogens *Staphylococcus, Pseudomonas, Escherichia/Shigella*, and *Enterococcus*. Microbial diversity differed significantly among the study samples, subcutaneous tissue, and positive/and negative controls (Supplementary Figure S1A). Six of eighteen analyzed devices had microbial growth on laboratory culture (Table 2). Overlap between the 16S raw sequences and the bacterial isolates cultured from the devices demonstrated that at least 10% of sequences represented viable bacteria (Supplementary Figure S1B). Microbial diversity did not differ by patient age at device removal/replacement (*p* = 0.27, Figure 2A), operative time for initial device placement (*p* = 0.26, Figure 2B), or device indwelling time (*p* = 0.56, Figure 2C).

### 3.2. Biofilm Metabolite Composition and Microbe–Metabolite Interaction Networks

**Figure 3:**
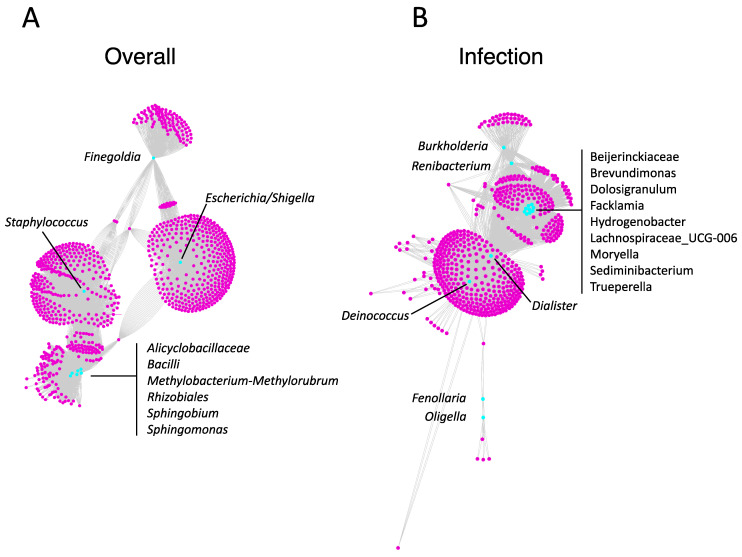
**Microbe–metabolite interaction networks on artificial urinary sphincter device biofilms.** (**A**) Microbe–metabolite interaction network based on relative counts for all analyzed artificial urinary sphincter devices. (**B**) Microbe–metabolite interaction network enriched in the presence of device-associated infection. Microbes are indicated in cyan and metabolites are indicated in magenta. Black lines connect microbes to respective metabolites. Genera are indicated in respective panels.

Metabolites detected on device biofilms are shown in Figure 1C. Identified metabolites with the greatest abundance included dimethylamine, 4r-aminopentanoic acid, ammonium acetate, 3-aminopropane-1,2 diol, and guanidine (comprehensive list in Supplementary Data Set 1). The overall microbial–metabolite network was developed based on microbe and metabolite counts for all analyzed devices (Figure 3A). The network revealed that the most central genera were *Staphylococcus* (2620 metabolites), *Escherichia/Shigella* (2101 metabolites), *Methylobacterium-Methylorubrum* (674 metabolites), *Bacilli* (506 metabolites), and *Finegoldia* (410 metabolites). A statistically significantly enriched subnetwork in the presence of infection was identified (Figure 3B). The most central genera in the infection subnetwork were *Dialester* spp. (1174 metabolites) and *Deinococcus* spp. (738 metabolites), followed by *Beijerinckiaceae* spp., *Brevundimonas* spp., *Dolosigranulum* spp., *Facklamia* spp., *Hydrogenobacter* spp., *Lachnospiraceae* spp., *Moryella* spp., *Sediminibacterium* spp., and *Trueperella* spp. (354 metabolites each) (Figure 3B).

### 3.3. Reconstitution of Bacterial Biofilm In Vitro

**Figure 4:**
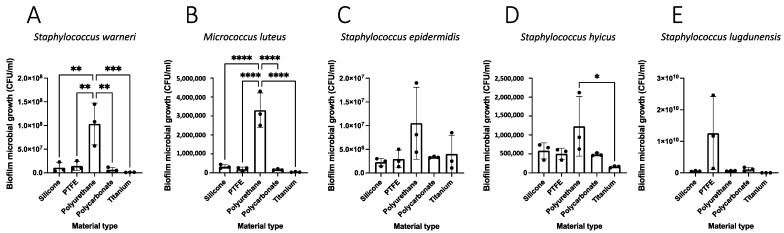
**Biofilm deposition of isolates obtained from indwelling artificial urinary sphincter devices varies by material type in vitro.** *Staphylococcus warneri* (**A**), *Micrococcus luteus* (**B**), *Staphylococcus epidermidis* (**C**), *Staphylococcus hyicus* (**D**), or *Staphylococcus lugdunensis* (**E**), were incubated together in a continuous-flow stir tank bioreactor with a series of coupons of different materials and assessed through plate count assays. Material types are indicated on the *x*-axis. Error bars indicate standard deviation. * *p* < 0.05, ** *p* < 0.01, *** *p* < 0.001, **** *p* < 0.0001.

**Figure 5:**
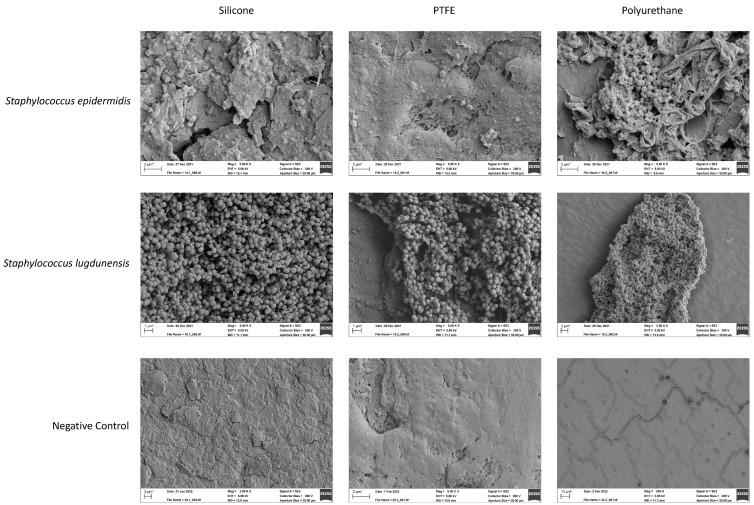
**Scanning electron microscopy shows visible biofilm formation across microbial strains.** Each strain was grown in a continuous-flow stir tank bioreactor with a series of coupons, which were subsequently analyzed using scanning electron microscopy. Microbial strains are indicated in row headings and material types indicated in column headings. Scale bars are indicated in the respective micrographs. Negative controls are shown in the final row. See Supplementary Figure S2 for all analyzed microbial strains and material types.

Analyzed isolates in the bioreactor were *Staphylococcus warneri*, *Micrococcus luteus*, *Staphylococcus epidermidis*, *Staphylococcus hyicus*, and *Staphylococcus lugdunensis*, and tested materials were silicone, polytetrafluoroethylene (PTFE), polyurethane, polycarbonate, and titanium. Negative controls for all material types, wherein coupons were incubated in the bioreactor with media alone, produced no growth. There was differential biofilm formation by material type for the majority of tested strains (Figure 4). Specifically, *S. warneri* (*p* < 0.01) and *M. luteus* (*p* < 0.0001) formed greater biofilm on polyurethane than on any other material type. *S. hyicus* formed more biofilm on polyurethane than titanium (*p* < 0.05). Scanning electron microscopy corroborated these results, demonstrating biofilm formation for all microbial strains. Visually, *S. lugdunensis* had the greatest biofilm deposition and polyurethane generally harbored robust biofilm (Figure 5, Supplementary Figure S2). 

### 3.4. Biofilm and Antibiotic Resistance Gene Detection

RT-PCR was performed on samples from 15 of the 18 device biofilms. Antibiotic resistance genes were commonly detected. Specifically, *sul2* (encoding for sulfonamide resistance) was detected in 8 of 15 (53%) biofilms. *ampC* (encoding for penicillin resistance) was detected in 11 of 15 (73%) biofilms. An *rpoB* mutation associated with rifampin resistance was detected in 8 of 15 (53%) biofilms. *tetA* (encoding for tetracycline resistance) was not detected in the biofilms. The *fimH* adhesin gene, associated with biofilm formation, was detected in 10 of 15 (67%) biofilms.

## 4. Discussion

We performed a detailed characterization of the biofilms present on artificial urinary sphincter devices. Using our multi-omics approaches, we have shown that, although 94% of analyzed devices in this study were not associated with infection, all devices unequivocally harbored biofilm. While many detected microbial genera were not typical pathogens, others including *Staphylococcus*, *Escherichia/Shigella*, and *Pseudomonas*, are known human pathogens that have been implicated in device-associated infection [5,42]. Antibiotic resistance genes were commonly detected on biofilms. Our data support a model wherein it is not the presence of a single organism that results in device-associated infection, but rather a dysbiotic shift, likely involving multiple organisms and their metabolites, that results in the transition from a colonized asymptomatic state to that of device-associated infection.

There is growing understanding of the pathophysiology of microbial adhesion and colonization of implanted medical devices [43,44]. Microbial contamination prior to or during surgical device implantation is a potential etiology of infection [43,45]. Open surgical wounds are contaminated with bacteria in the planktonic, free-floating form, and in most cases the body’s defenses, together with antibiotic prophylaxis, prevent against infectious sequelae. However, in cases wherein a foreign body, such as an AUS device, is implanted, the body rapidly coats the surface of the material with a conditioning layer comprised of albumin, fibrinogen, and other proteins, thereby altering the device’s surface [43,44,46]. The conditioning layer facilitates attachment by both immune cells and planktonic bacteria in what has been described as a “race to the surface” [44]. Following initial microbial adhesion, biofilm formation may ensue. 

In contrast to free-floating planktonic bacteria, biofilms are communities of bacteria adherent to one another and a surface, and encased in extracellular matrix. Biofilms allow for immune evasion and are responsible for the persistence of microbial infections [44]. Thus, implant-associated infections usually require surgical removal. Biofilms can tolerate 10–1000-fold higher antibiotic concentration relative to microbes in the planktonic state [43]. Further, biofilms allow for the efficient horizontal gene transfer of antimicrobial resistance [47], and can serve as a nidus for bacterial dissemination to distant anatomical sites.

Preventative strategies have been implemented to reduce the risk of AUS infection [46]. One example is the optimization of sterile skin prep formulation. In a randomized controlled clinical trial, in those undergoing clean-contaminated surgery, a chlorhexidine–alcohol scrub resulted in a significantly lower infection rate relative to povidone–iodine scrub [48]. In another randomized controlled trial of those undergoing genitourinary prosthetic surgery, chlorhexidine–alcohol was superior to povidone–iodine in the eradication of skin flora at the surgical site [49]. Whether this translates to improved clinical outcomes in this population remains to be determined. Although those with preoperative positive urine cultures are often treated with antibiotics prior to surgery, evidence suggests that this may not improve outcomes [50,51]. Interestingly, there was only a 7% correlation between organisms isolated on preoperative urine culture and those isolated in the context of device-associated infection [51]. In the present study, all AUS devices harbored microbiota, even in the absence of infection. Future work to identify the microbes and metabolites involved in the shift from the colonized (asymptomatic) state to the infected state is needed, and to optimize the next generation of antimicrobial agents and medical device materials to reduce infection risk. The present study provides the groundwork for such investigation.

The most commonly detected phyla in AUS in the present study were Proteobacteria, Firmicutes, Actinobacteriota, and Actinobacteria. The Proteobacteria, Firmicutes, and Actinobacteria are also three of the four predominant gut microbiota phyla [52]. The possibility that, and mechanism by which, gut flora gain access to indwelling AUS devices warrants investigation. Potential avenues include direct inoculation during the index surgery and extravasation from the gut to the subcutaneous tissue over time, perhaps facilitated by frequent pump manipulation. Given that these flora were commonly present even in the absence of infection, it also remains to be determined whether prevention of such biofilm formation would reduce the risk of infection. It is more likely that targeted microbial pattern alteration could reduce infection risk. How the device microbiome may be intentionally modulated to that end is an area primed by the present study for future investigation. 

The most commonly detected metabolites identified included dimethylamine, 4r-and aminopentanoic acid. Diethylamine is a metabolic breakdown product of *Micrococcus* spp. [53] (Figure 1B). Aminopentanoic acid is a potential bacterial metabolite of *Pseudomonas* spp. [54,55]. Both *Micrococcus* spp. and *Pseudomonas* spp. were detected in biofilms by the next-generation sequencing approach. We suspect these microbes and metabolites may be of importance in the transition from colonization to infection. Indeed, one study found that *Pseudomonas* spp. was isolated from 9% of AUS devices that were explanted for infection [42].

Inhibizone, a coating consisting of rifampin and minocycline, has been shown to reduce infection risk when applied to penile prosthesis devices [56]. However, studies suggest that Inhibizone coating on AUS devices is not associated with a reduction in infection [1,4]. In the present study, we detected the presence of the *rpoB* mutation, which is associated with rifampin resistance, in biofilms of 8 of 15 (53%) analyzed devices. One of two possible scenarios may have accounted for this finding. First, the mutations may have been present in the microbes prior to device colonization. In this case, a rifampin coating would not be efficacious against bacteria harboring this mutation. Second, rifampin coating on AUS devices may have selected for rifampin resistance. While these data do not directly address the efficacy of the Inhibizone coating, they call for further investigation regarding the risk of resistance selection relative to any benefit of reduction in infection, and how this differs between AUS devices and penile prostheses. 

The generalizability of the findings in AUS devices in the present study to penile prostheses and other device types is not yet understood. Potential mechanistic differences for microbial colonization between these device types include incision proximity to the rectum during index surgery (the AUS device perineal incision is generally more proximal to the rectum), and a potentially higher propensity for bacterial translocation across the urethra in AUS devices. It is also plausible that there is a contribution of seeding from urinary microbes during index surgery, which may differ by device type.

We used a biofilm reactor assay that is designed to mimic a medical device present in human tissue. The model allows for the assessment and comparison of different device materials and microbial strains for biofilm formation in vitro [41]. In this study, we demonstrated differences in biofilm deposition by material type. Specifically, polyurethane had the greatest biofilm deposition by *Staphylococcus warneri* and *Micrococcus luteus*. *Staphylococcus epidermidis*, which is among the most common causative microbes of AUS infection [42], exhibited no difference in biofilm deposition among the five materials studied. There were no differences detected among silicone, PTFE, polycarbonate, and titanium across strains. These device materials are used not only in continence devices, but also across other urologic devices and other medical devices used in diverse surgical disciplines. The optimal device type will be resistant to infection while having excellent durability and the malleability needed for a continence mechanism. While silicone, the AMS 800 material, is durable and malleable, it remains subject to infection in a subset of cases, and thus novel material types in tandem with coatings or other anti-infective strategies are needed. The bioreactor assay described here is poised to screen for such materials.

The limitations of this study include its relatively small sample size and the inability to detect fungi or viruses in biofilms. Despite this, our study has several strengths. Strengths include the use of a multi-omics approach, wherein we provide an in-depth characterization of the microbes, metabolites, and their interaction networks within the AUS biofilm. Second, the study uses a multimodal approach, including plate count assays and scanning electron microscopy, to demonstrate the biofilm formation capacity of isolated bacteria across materials. The assay described is versatile and may be adapted to screen for novel materials and coatings to reduce infection risk on AUS devices, but also other implanted medical devices including sacral neuromodulation devices, joint prosthetics, permanent pacemakers, and others. 

## 5. Conclusions

All artificial urinary sphincter devices harbored microbial biofilms, even in the absence of infection. Specific microbes, metabolites, and their interaction networks were identified within biofilms. Antibiotic resistance genes, including a mutation that confers rifampin resistance, were commonly detected in biofilms. The tested microbial strains, including *Staphylococcus epidermidis*, reconstituted biofilm across material types in vitro in an assay mimicking subcutaneous tissue with medical device implant. Polyurethane material harbored the greatest biofilm deposition for *Staphylococcus warneri* and *Micrococcus luteus.* Silicone, PTFE, polycarbonate, and titanium harbored no difference in biofilm formation in all studied strains. The results of this investigation, wherein we comprehensively characterized the composition of AUS device biofilms, provide the framework for future identification and rational development of inhibitors and preventive strategies against device-associated infection.

## Figures and Tables

**Table 1:** Demographics from individuals from whom artificial urinary sphincters were analyzed.

Factor	Mean (SD) or *n* (%) (*n* = 18)
Age (years)	73 (10.2)
Sex (male)	18 (100)
Race	
–White	15 (83.3)
–Black	2 (11.1)
–Hispanic	1 (5.6)
Body mass index (kg/m^2^)	28.9 (5.1)
Diabetes mellitus	2 (11.1)
Cardiac disease	6 (33.3)
Current smoker	0 (0)
Device indwelling time (years)	6.4 (4.8)
Operative time at placement (minutes)	115 (47.1) ^1^
Indication for device removal	
–Device malfunction	13 (72.2)
–Device-associated infection	1 (5.6)
–Cuff erosion	3 (16.7)
–Clinically ineffective	1 (5.6)
Antibiotics during 30 days prior to device removal	6 (33.3)

^1^ Operative time of initial placement unknown for four individuals.

**Table 2:** Cultured and identified microbial strains isolated from artificial urinary sphincters.

Strain ^1^	Number of Isolates
*Staphylococcus epidermidis*	2
*Staphylococcus lugdunensis*	2
*Staphylococcus hyicus*	1
*Staphylococcus warneri*	1
*Micrococcus luteus*	1
*Micrococcus yunnanensis*	1
*Enterococcus faecalis*	1
*Brevibacterium* sp	1

^1^ Swabs were taken from devices of all 18 patients. A total of 10 strains were isolated and identified (6 unique patients). Six of eighteen devices had growth on culture (33.3%).

## Data Availability

Because research subjects did not provide consent for public sharing of data, data sharing is not available for this study.

## Supplementary Materials

The following supporting information can be downloaded at: https://www.mdpi.com/article/10.3390/biomedicines11010215/s1, Supplementary Figures S1 and S2, Supplementary Data Set.
