# Supplementary material for: Biofilms on Indwelling Artificial Urinary Sphincter Devices Harbor Complex Microbe–Metabolite Interaction Networks and Reconstitute Differentially In Vitro by Material Type
Source: Biomedicines. 2023 Jan 14;11(1):215. doi: 10.3390/biomedicines11010215 (PMC9855829; doi:10.3390/biomedicines11010215)
Supplement: Supplementary file 1 [file biomedicines-11-00215-s001.zip › biomedicines-2150190-supplementary.pdf]

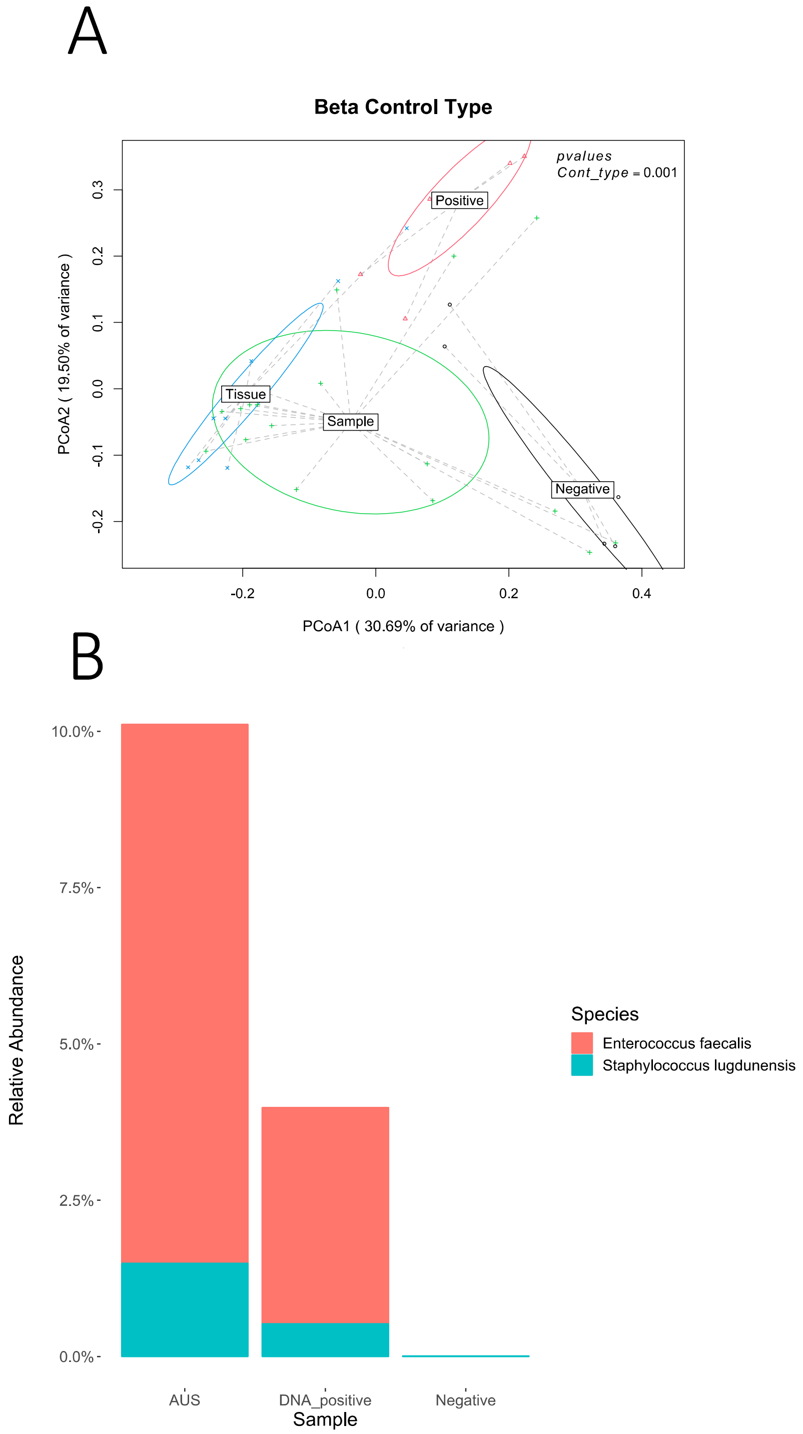

Supplementary Figure S1: Bioinformatic controls. Beta diversity (A) by sample type. P-value is indicated in the panel. Positive controls include commercial DNA standards and a laboratory cultured urinary bacteria. Negative controls included DNA extraction reagents, and a PCR negative control. Tissue refers to subcutaneous tissue. (C) Mapping of 16s sequencing to sequencing of cultured isolates reveals overlap, suggesting at least 10% of sequences were derived from viable bacteria.

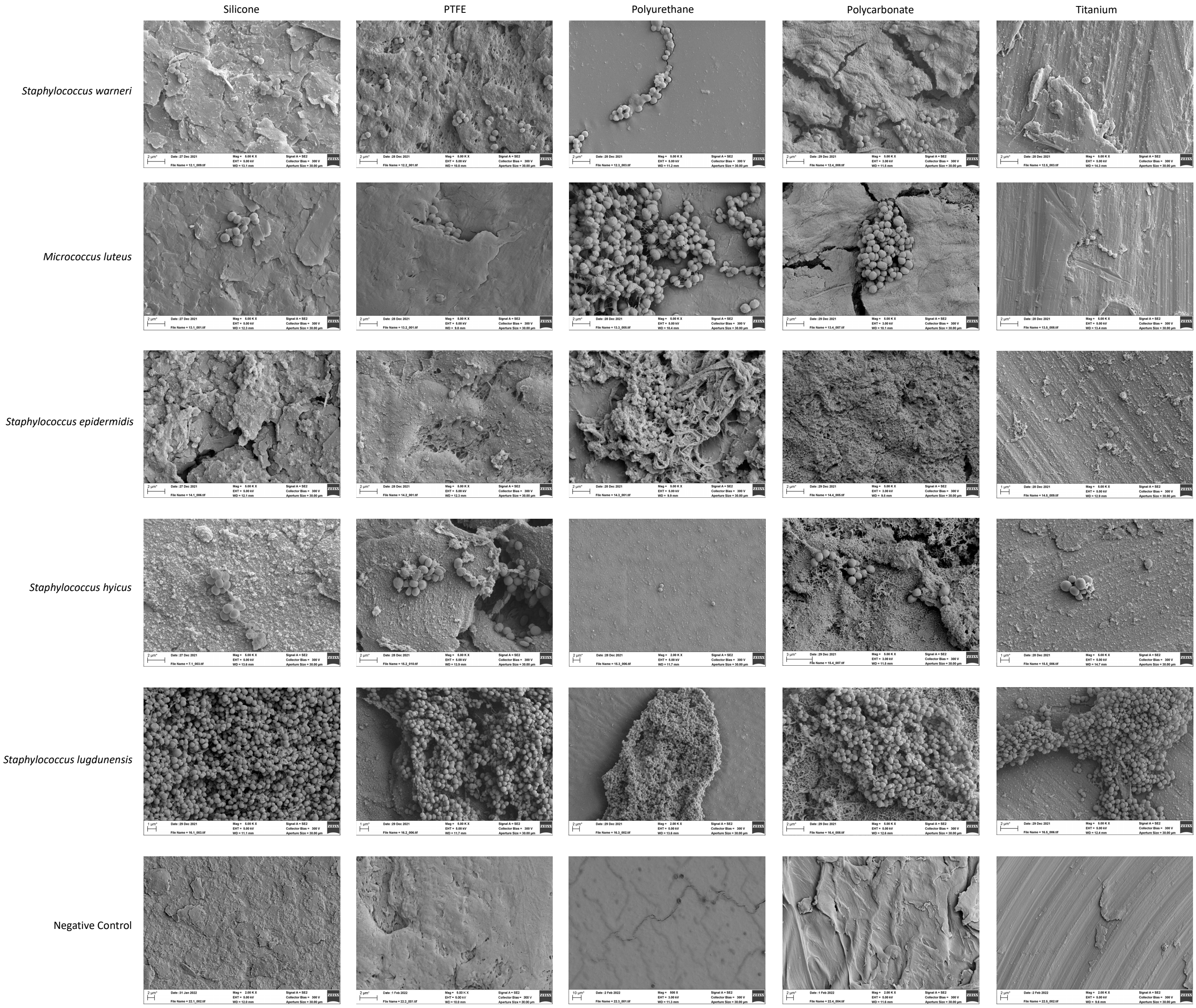

Supplementary Figure S2: Scanning electron microscopy shows visible biofilm formation across microbial strains. Each strain was grown in a continuous-flow stir tank bioreactor for 72 hours along with a series of coupons or different material types. Coupons of silicone, PTFE, polyurethane, polycarbonate, and titanium were rinsed of nonadherent bacteria, fixed, mounted, sputter coated, and imaged. Microbial strains are indicated in row headings and material types indicated in column headings. The negative control, wherein coupons were incubated in the bioreactor under identical conditions for 72 hours in sterile media, is shown in the final row. Scale bars are indicated in the respective micrographs.

Supplementary Data Set

**Metabolite**	**Average**
Other	0.27781048
dimethylamine	0.15590371
unknown	0.06313953
4r-aminopentanoic acid[m+acn+h]	0.04557947
ammonium acetate[m+h-h2o]	0.02361083
3-aminopropane-1,2-diol[m+h-h2o]	0.01959868
guanidine[m+h]	0.01723361
carbachol[m+h-2h2o]	0.0167408
phosphoric acid [m-h]	0.01542502
<[m+acn+h]+>	0.01542459
trans,trans-muconic acid [m-h]	0.01427922
acetic acid	0.01350871
nervonyl carnitine[m+acn+h]	0.0117457
magnesium dipropionate	0.01163123
<[m+h-2h2o]+>	0.01041881
1h-imidazole, 1-butyl-2-methyl-[m+h]	0.00949615
ribitol	0.00942352
<[m+h]+>	0.009056
1-tetrahydro-3-furanyl-4-piperidinamine [m+h]	0.00903344
3-methylamino-1,2-propandiol[m+h-2h2o]	0.00860805
1-pentanethiol[m-k-2h]	0.0076649
1h-indole-3-carboxylic acid, 1-[2-(2,5-dimethylphenoxy)ethyl]- [m+h-h2o]	0.00741732
1,2-dichloroethane[m+acn+h]	0.00721134
5alpha-cholane[m+h-h2o]	0.00718215
6-fluoro-4-hydroxycoumarin [m+h]	0.00677102
gamma-coniceine[m+acn+h]	0.00651933
glycolic acid tetraethoxylate lauryl ether [m+h]	0.00650481
tetrahydroxypteridine[m-k-2h]	0.00644726
.epsilon.-caprolactam [m+h]	0.00619889
polypropylene glycol (m w 1,200-3,000)[m+acn+h]	0.0059848
aragusteroketal[m+acn+h]	0.00567993
<[m-h2o-h]->	0.00557318
acetyl phosphate[m+hac-h]	0.00555452
2-amino-3-methyl-1-butanol[m+h-h2o]	0.00540346
<phe ile ile thr[m-h2o-h]->	0.00488427
1,4-dihydroxy-6,6,9a-trimethyl-4,5,5a,6,7,8,9,9a-octahydronaphtho[1,2-c]furan-3(1h)-one[m-h]	0.00443544
3-hexanoyl-nbd cholesterol[m+h]	0.00440383
2-thiophenemethanethiol[m-h2o-h]	0.00409235
1-aminocyclobutane carboxylic acid[m+acn+h]	0.00390392
p-cresol sulfate[m-h2o-h]	0.00376532

dipicolinic acid [m-h]	0.00363363
creatine[m+h-h2o]	0.00359608
<lys lys arg thr[m+h]	0.00349488
dimetacrine tartrate[m+h]	0.00348163
bis (2-hydroxypropyl) amine[m+h]	0.00346356
7-chloroemodin[m+h-2h2o]	0.00315487
citric acid[m-h2o-h]	0.00309902
5-methylcytosine[m-h2o-h]	0.00309431
<0[m+h-2h2o]+>	0.00307743
diisopropylethylamine [m+h]	0.0028367
<[m-h]->	0.00282989
11-amino-undecanoic acid[m+h]	0.00282828
2-imino-4-methylpiperidine [m+h]	0.00281785
2,4-dichlorotoluene[m-cl]	0.00278091
4-bromophenylacetate[m-h2o-h]	0.00264584
3,6-dihydroxycyclohexane-1,2,4,5-tetraone	0.00261088
(-/-)-3-[(2-methyl-3-furyl)thio]-2-butanone[m-h2o-h]	0.00259754
sannamycin a[m-fa-h]	0.00251794
anthenoside a[m+h]	0.00248559
lactic acid[m-na-2h]	0.00246529
ammonium isovalerate[m+h-h2o]	0.002421
metapramine [m+h]+>;	0.00241543
<[m+h-h2o]<[m+acn+h]+>	0.00237825
1-methylphenanthrene[m+h-h2o]	0.00223918
2-chlorophenylhydrazine hydrochloride[m-cl]	0.00206571
antibiotic x 14889d[m+h-2h2o]	0.00205198
lawsone methyl ether[m-cl]	0.00203867
p32/98 hemifumarate salt[m+h-h2o]	0.00200725
ps(o-16:0/15:0)[m+h]	0.00200381
2-aminobiphenyl [m+h]	0.00199146
<lysopc(24:1(15z))[m+hac-h]	0.00198146
<dg(20:4(5z,8z,11z,14z)/20:5(5z,8z,11z,14z,17z)/0:0)[iso2][m+hac-h]	0.0018799
myristoyl-l-carnitine [m+na]+>	0.00186227
didodecyl 3,3'-thiodipropionate [m+h]	0.00185306
tubaic acid	0.0018144
salicylic acid[m-h2o-h]	0.00178974
2-methoxymethcathinone [m+h]	0.00175163
dl-normetanephrine [m+h-h2o]	0.00165017
12-amino-dodecanoic acid[m+acn+h]	0.00163964
embelin[m-h]	0.00163528
<0[m+acn+h]+>	0.00159229
p-chloroacetoacetanilide[m-h2o-h]	0.00157553
trimethylaminoacetone[m+acn+h]	0.00155169
cetrimonium[m+h]	0.0014987
1,3,5-trithiane[m-na-2h]	0.00149366

thr lys lys[m-h]	0.00149122
1,2-bis(chloromethoxy)ethane[m+acn+h]	0.00147841
<[m+hac-h]<[m+hac-h]+>	0.00138255
palmitoylcarnitine [m+na]+>	0.00138141
beta-cymaropyranose[m-na-2h]	0.00134668
2,4(1h,3h)-pyrimidinedithione	0.00125771
lidocaine [m+h]	0.00115622
hexadecanoic acid [m-h]	0.00110765
2-isopropyl-n-methylaniline [m+h]	0.00106523
sant-1[m+hac-h]	0.00105125
1-phenylethylamine[m+h]	0.00101041
1-butanethiol[m-k-2h]	0.00085626
iso-olomoucine [m+h-h2o]	0.00083799
3,4,5-trimethoxy-n-3-piperidinylbenzamide [m+h]	0.00076157
4-amino-4-methylpentanoic acid [m+h]	0.00064894
betaine aldehyde[m+acn+h]	0.0006241
l-carnitine [m+na]	0.00056188
glucoheptonic acid[m-h]	0.00049072
penciclovir[m+h-h2o]	0.00044052
l-2-aminobutyric acid [m+h]	0.00038213
5-amino-2-methoxyphenol [m+h]	0.00019928
nitric acid [2m-2h+na]	0.00019886
n-hexanoyl-dl-homoserine lactone [m+h]+>;	0.00019052
3,4-diphenyl-7-hydroxycoumarin [m-h]	5.81E-05
d-desthiobiotin	4.17E-05
norpropoxyphene [m+h]+>;	1.12E-05
bupivacaine [m+h]	7.57E-06
4-hydroxy-n-methyl-n-isopropyltryptamine [m+h]	0
